# Turning Antibodies into Ratiometric Bioluminescent Sensors for Competition-Based Homogeneous Immunoassays

**DOI:** 10.1021/acssensors.3c02478

**Published:** 2024-02-21

**Authors:** Eva A. van Aalen, Joep J. J. Lurvink, Leandra Vermeulen, Benice van Gerven, Yan Ni, Remco Arts, Maarten Merkx

**Affiliations:** † Laboratory of Chemical Biology, Department of Biomedical Engineering, Eindhoven University of Technology, P.O. Box 513, Eindhoven 5600 MB, The Netherlands; ‡ Institute for Complex Molecular Systems, Eindhoven University of Technology, P.O. Box 513, Eindhoven 5600 MB, The Netherlands

**Keywords:** BRET, biosensors, competition assay, antibodies, homogeneous immunoassay, small molecules

## Abstract

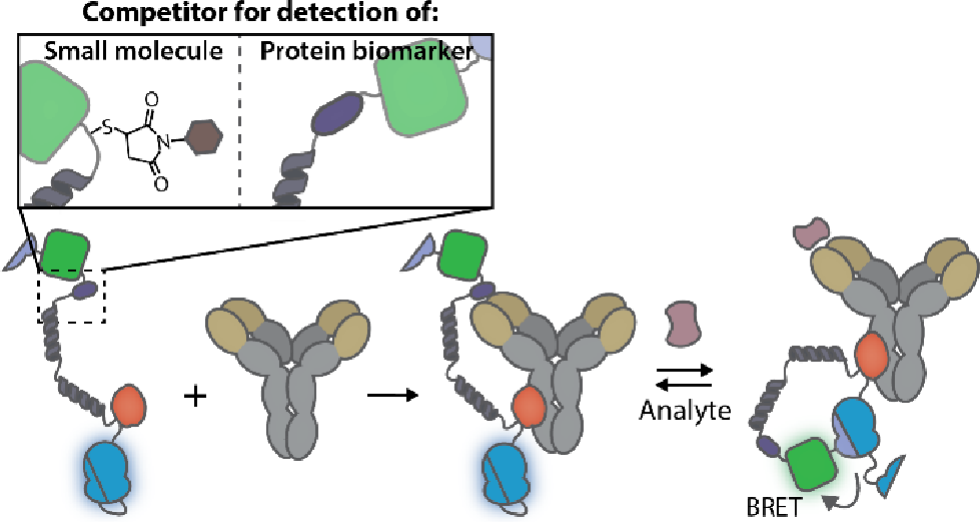

Here we present LUCOS (Luminescent Competition Sensor), a modular and broadly applicable bioluminescent diagnostic platform enabling the detection of both small molecules and protein biomarkers. The construction of LUCOS sensors entails the covalent and site-specific coupling of a bioluminescent sensor component to an analyte-specific antibody via protein G-mediated photoconjugation. Target detection is accomplished through intramolecular competition with a tethered analyte competitor for antibody binding. We established two variants of LUCOS: an inherent ratiometric LUCOSR variant and an intensiometric LUCOSI version, which can be used for ratiometric detection upon the addition of a split calibrator luciferase. To demonstrate the versatility of the LUCOS platform, sensors were developed for the detection of the small molecule cortisol and the protein biomarker NT-proBNP. Sensors for both targets displayed analyte-dependent changes in the emission ratio and enabled detection in the micromolar concentration range (*K*_D,app_ = 16–92 μM). Furthermore, we showed that the response range of the LUCOS sensor can be adjusted by attenuating the affinity of the tethered NT-proBNP competitor, which enabled detection in the nanomolar concentration range (*K*_D,app_ = 317 ± 26 nM). Overall, the LUCOS platform offers a highly versatile and easy method to convert commercially available monoclonal antibodies into bioluminescent biosensors that provide a homogeneous alternative for the competitive immunoassay.

The ability to generate specific antibodies against almost any relevant biomolecule renders immunoassays widely applied bioanalytical tools for the detection of both protein biomarkers and small molecules. The heterogeneous enzyme-linked immunosorbent assay (ELISA) is routinely used in clinical laboratories, and several ELISA variants have been established to enable the sensitive detection of a broad range of relevant targets.^1^ For example, the sandwich ELISA, based on capturing a target analyte by two distinct antibodies, is often applied to measure protein biomarkers, whereas competitive immunoassays are commonly employed for small molecule quantification.^2^ However, the development of an ELISA is not straightforward, as it involves the optimization of a number of aspects such as surface immobilization and careful consideration of assay conditions to minimize background binding.^2,3^ Furthermore, its intrinsic complex, multistep workflow hampers usage outside of traditional laboratories and at the point-of-care (POC).

Homogeneous biosensors based on a bioluminescent readout show great potential for applications in POC settings because they do not require external illumination and allow one-step, in-sample measurements.^4^ We recently established the RAPPID platform, a highly modular bioluminescent immunoassay that enables the detection of a wide variety of clinically relevant protein biomarkers.^5−7^ These sensors comprise of split NanoLuc (NLuc) luciferases covalently coupled via protein G adapters to antibodies, where target-induced complementation of these split NLuc fragments causes an increase in bioluminescent signal.^8−10^ The RAPPID platform can be readily adapted for the quantification of new protein biomarkers, simply by exchanging the target-specific antibodies in the assay. Furthermore, the intrinsic homogeneous nature and bioluminescent readout of RAPPID expands its application beyond traditional laboratories and renders RAPPID particularly suitable for measurements in POC settings. However, RAPPID does not enable the detection of small molecules, due to the requirement of two antibodies binding to distinct epitopes on the target analyte.

Several classes of (bioluminescent) biosensors dedicated to small molecule sensing have been developed,^11−14^ including the luciferase-based indicators of drugs (LUCIDs), introduced by Johnsson and coworkers. The first generation of LUCID sensors depended on small molecule recognition by an analyte-specific receptor domain and entailed competition between a tethered competitor ligand and the free target small molecule for binding to this receptor-binding domain.^15^ However, the reliance on a suitable receptor reduced the number of potential targets. Therefore, Xue et al. next established more generic LUCID variants with antigen-binding (Fab) fragments of antibodies as binding domains.^16^ A second platform of bioluminescent immunosensors that enable the detection of small molecules is the bioluminescence resonance energy transfer (BRET)-based Q-bodies (quenchbodies) developed by Ueda and coworkers.^17^ This sensor format comprises of an analyte-specific single-chain antibody (scFv) fragment that is genetically fused to NLuc, and subsequently labeled with a fluorescent dye. Here, the presence of target analyte induces a change in BRET-efficiency due to the release of a quenched fluorophore from the scFv fragment. While LUCIDs and Q-bodies have been successfully developed for a range of small molecules, the development of a sensor for a new analyte still requires the cloning and expression of new fusion proteins that incorporate target-specific Fab or scFv fragments.

Here we introduce LUCOS (Luminescent Competition Sensor), a modular sensor platform that involves tailoring of monoclonal antibodies to yield BRET-based immunosensors for the detection of both small molecules and protein biomarkers. Similar to the plug-and-play RAPPID, LUCOS does not require the genetic incorporation of a target binding domain, rendering the sensor platform highly adaptable and suitable for the detection of a wide range of biomolecules. LUCOS comprises of a bioluminescent sensor component coupled to an analyte-specific antibody through protein G-mediated photoconjugation.^10^ The sensing concept is based on competition between an intramolecular analyte ‘competitor’ and the free target of interest for binding to the antigen binding domain of the antibody (Figure 1a,c). Displacement of the competitor from the binding site of the antibody by the target analyte induces the closed state of the sensor, resulting in an analyte-dependent change in the bioluminescent emission ratio (Figure 1b,d). We developed two variants of LUCOS: an inherent ratiometric (LUCOSR) version and an intensiometric (LUCOSI) version, with a ratiometric output upon the addition of a split calibrator luciferase (Figure 1a,c, respectively). We subsequently demonstrated their potential for the quantification of both small molecules and protein biomarkers by developing sensors for the clinically relevant hormone cortisol and the NT-proBNP peptide.

**Figure 1:**
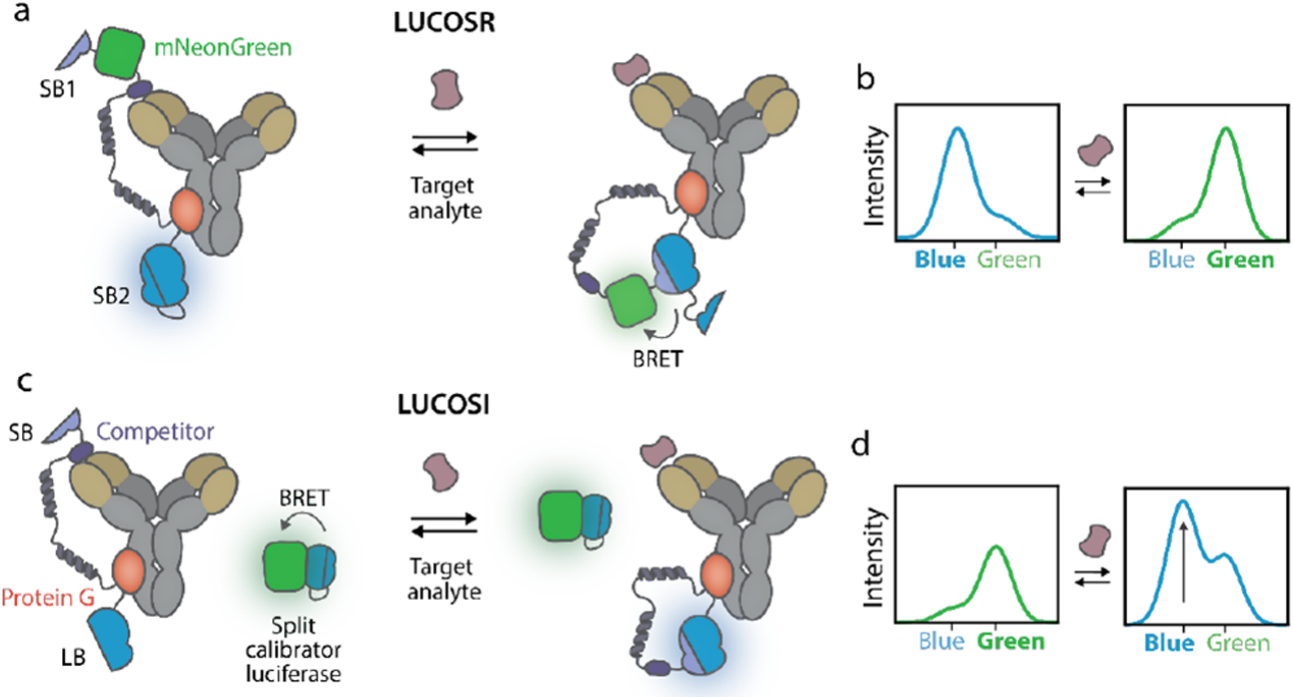
General overview of the LUCOS platform. (a) Schematic representation of the ratiometric LUCOS (LUCOSR). Binding of the competitor to the antigen-binding site of the antibody engenders the open sensor conformation and allows the low-affinity SB2 to bind to the LB. Upon the addition and binding of the target analyte, SB2 is displaced by a higher affinity SB1, bringing mNeonGreen close to the reconstituted NLuc and enabling efficient BRET. (b) The change in the bioluminescent output of the LUCOSR sensor in the presence of the target analyte. (c) Schematic illustration of the intensiometric LUCOS (LUCOSI). The target-specific antibody is covalently coupled to the LUCOSI sensor domain via protein G-mediated photoconjugation. The sensor adopts an open conformation in the absence of the target analyte due to the binding of an analyte–competitor to the antibody. This results in the spatial separation of small BiT (SB) and large BiT (LB) and a low luminescent signal. In the presence of the target analyte, the competitor is displaced, allowing the reconstitution of NLuc and the emission of blue light. To provide for robust ratiometric sensor outputs, the split calibrator luciferase is added to the assay, enabling in-sample calibration. (d) The sensor output of LUCOSI, with the split calibrator luciferase, goes from green to blue in the presence of the target analyte.

## Experimental Section

### Cloning

The LUCOS sensor constructs for the detection of NT-proBNP were obtained from the pET28a(+)-LUCOSI and LUCOSR plasmids (Figures S12 and S13) using standard cloning techniques (Figures S14 and S15). The correct sequences of the expression plasmids were verified using Sanger dideoxy sequencing.

### Protein Expression

The pET28a(+)-LUCOSI and LUCOSR plasmids (Figures S12–S15) were cotransformed in *Escherichia coli* (*E. coli*) BL21 (DE3) together with a pEVOL vector encoding a tRNA/tRNA synthetase pair for the incorporation of the unnatural amino acid para-benzoyl-phenylalanine (*p*BPA) at the amber stop codon.^18^ The pEVOL vector was a gift from Peter Schultz (Addgene plasmid # 31 190). Subsequently, the cells were cultured at 37 °C in LB medium (25 g/L) containing 50 μg/mL kanamycin and 30 μg/mL chloramphenicol until an OD600 of 0.6 was reached. Next, 0.1 mM IPTG and 0.2% arabinose, in the presence of 1 mM *p*BPA (Bachem, 104 504–45–2), were added to the LB medium to induce protein expression. After 12 h of incubation at 18 °C (shaken at 180 rpm), the cells were harvested by centrifugation at 10 000 *g* for 10 min and subsequently lysed with Bugbuster and Benzonase (both from Novagen). The lysed bacteria were centrifuged at 16 000 *g* for 45 min at 4 °C and purified from the supernatant using Ni^2+^ affinity chromatography followed by Strep–Tactin purification according to the manufacturer’s instructions. Subsequently, the absorbance of the proteins at 280 nm (measured using a NanoDrop spectrophotometer ND-1000) and the extinction coefficients of the proteins were used to determine the concentrations of the expressed proteins and the split calibrator luciferase. Proteins were stored at −70 °C, and prior to photoconjugation or labeling, they were diluted to working concentrations. The purity of the LUCOS-base proteins was determined using SDS-PAGE analysis (Figures S3 and S9), and the incorporation of *p*BPA was confirmed by Q-ToF LC-MS (WatersMassLynx v4.1), using MagTran v1.03 for MS deconvolution.

### Cortisol-3-CMO-Maleimide Synthesis

Ten mg hydrocortisone 3-(O-carboxymethyl)oxime (Toronto Research Chemicals) and 10.4 mg 2-(7-Aza-1H-benzotriazole-1-yl)-1,1,3,3-tetramethyluronium hexafluorophosphate (HATU, Fluorochem) were dissolved in 2 mL DMF. Subsequently, 44 μL diisopropylethylamine (DIPEA, Sigma) was added together with 16.5 mg 1-(2 aminoethyl)maleimide hydrochloride (Sigma) before the mixture was stirred for 4 h at room temperature. DMF was removed using a Buchi rotary evaporator and a diaphragm pump. The remaining compound was dissolved in Milli-Q water with 25% HPLC-grade acetonitrile and 0.1% formic acid. Cortisol-3-CMO-maleimide was purified with a preparative LC-MS system comprising of an LCQ Deca XP Max (Thermo Finnigan) ion-trap mass spectrometer equipped with a Surveyor photodiode detector array (PDA) detector (Thermo Finnigan). The mixture was purified with a Phenomex kinetex 2.6 μm EVO C18 50 × 2.1 mm column and an isocratic acetonitrile gradient of 34%. Fractions with the correct mass (E/Z isomers were not separated) were collected using a PrepFC fraction collector (Gilson Inc.) and subsequently freeze-dried before being dissolved in DMSO and stored at −30 °C (Figure S4).

### Maleimide–Competitor Coupling

**Figure 2:**
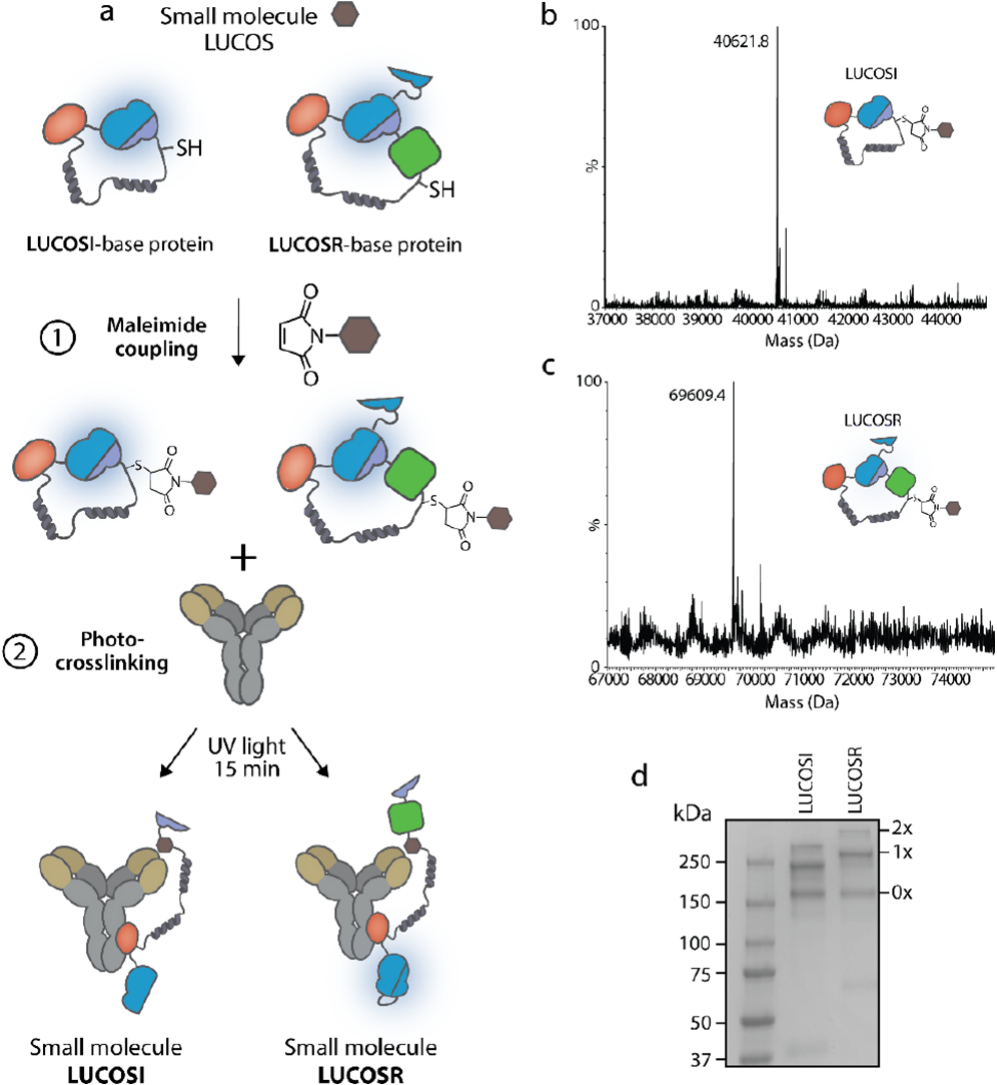
Construction of LUCOS sensors for cortisol sensing. (a) Schematic overview of the development of the LUCOSI and LUCOSR for the detection of small molecules. After recombinant expression in *E. coli*, the sensor proteins are (1) labeled with a maleimide–analyte–competitor via maleimide-cysteine chemistry and (2) photo-crosslinked to a target-specific antibody via protein G-mediated photo-crosslinking. Photo-crosslinking was done in PBS (pH 7.4) for 15 min under ultraviolet (UV) light (λ = 365 nm). ESI-QToF mass spectra of (b) LUCOSI coupled to cortisol-3-CMO–malemide (calculated mass without the N-terminal methionine: 40 620.6 Da) and (c) LUCOSR (calculated mass without the N-terminal methionine: 69 607.1 Da). (d) Nonreducing SDS-PAGE analysis of the photoconjugation between the cortisol-labeled LUCOS and an anticortisol antibody, in a 1:1 molar ratio.

First, 50 μM of purified LUCOS-base proteins were diluted in 1 mL 100 mM sodium phosphate buffer (pH 7) and reduced with 5 mM tris(2-carboxyethyl)phosphine (TCEP) for 1 h at room temperature (shaking incubator 500 rpm). Next, to remove the excess of TCEP, the sample was loaded onto a PD-10 desalting column (Cytvia) that was first equilibrated with 100 mM sodium phosphate buffer (pH 7) with 25 μM TCEP. For the maleimide coupling reaction, the reduced LUCOS-base protein was mixed with the cortisol-3-CMO-maleimide in a 1:10 molar ratio (7.5 μM sensor protein with 75 μM cortisol-3-CMO-maleimide) in 100 mM sodium phosphate buffer (pH 7, with 5% DMSO). The reaction mixture was subsequently incubated for 2 h at room temperature (500 rpm). Next, a PD-10 desalting column was used to remove excess, unreacted cortisol-3-CMO-maleimide and exchange the reaction buffer with PBS (pH 7.2). Successful incorporation of the analog was confirmed with Q-ToF LC-MS (Waters MassLynx v4.1), using MagTran v1.03 for MS deconvolution (Figures 2b,c and S5). Proteins were stored at −70 °C until use.

### Photoconjugation

Monoclonal mouse IgG2a cortisol antibody ab1949 (XM210) was obtained from Abcam, and monoclonal mouse IgG2a proBNP antibody NB120–14712 (13G12cc) was purchased from Novus Biologicals. The LUCOS sensor components (1 μM) and antibody (1 μM) were mixed in PBS (pH 7.4) in a final volume of 20 μL and preincubated for 30 min at room temperature. Photoconjugation reactions were subsequently performed using a UV lamp (Thorlabs M365LP1 with a Thorlabs LEDD1B T-Cube LED Driver) for 15 min.^6^ After conjugation, the LUCOS sensors were not further purified and stored at 4 °C until use.

### Luminescent LUCOS Assays

Cortisol was obtained from Sigma, and the NT-proBNP immunogenic peptide (ETSGLQEQRNHLQGK) used for the bioluminescence titrations was ordered from Genscript and dissolved in Milli-Q water (Figures S14 and S15). Luminescent assays were performed in NUNC white 384 flat well plates with 250 pM LUCOSI or LUCOSR for the cortisol measurements and 250 pM or 100 pM for the NT-proBNP titrations, respectively. Furthermore, 50 pM split calibrator luciferase was added to the intensiometric LUCOS measurements. The DNA sequence and expression and purification method of the split calibrator luciferase are described in ref^19^. The LUCOS sensor, split calibrator luciferase, and the target analyte were mixed in PBS buffer (pH 7.4, 0.1% (w/v) BSA), with 5% dimethyl sulfoxide (DMSO) for cortisol measurements, and incubated for 2 h at room temperature. Measurements in human blood serum (Sigma-Aldrich, H4522) were performed in PBS buffer (pH 7.4, 0.1% (w/v) BSA, 5% DMSO) with 20% human serum. Subsequently, NLuc substrate furimazine (Promega, N1110) was added with a final 1000-fold dilution, and the luminescent spectra were recorded between 398 and 653 nm with an integration time of 0.5 s using a Tecan Spark 10 M (bandwidth 25 nm; room temperature). The green-to-blue ratios were obtained by dividing the green light emission at 518 nm by the blue light at 458 nm.

## Results and Discussion

### LUCOS Sensor Design

The LUCOSI sensor component comprises of a protein G adapter (Gx), which can be photo-crosslinked to the constant Fc domain of antibodies via the non-natural amino acid *p*-benzoylphenylalanine, genetically fused to the large BiT (LB) fragment and the small BiT (SB, *K*_D_ = 2.5 μM) peptide of split NLuc (Figure 1c).^8−10^ We selected an SB with a *K*_D_ = 2.5 μM because thermodynamic modeling suggested that the affinity of this SB variant enables analyte detection in the nanomolar-to-micromolar concentration range with relatively large changes in NLuc emission (Figures S1 and S2a). The LB protein is connected to the Gx domain via a short GGS linker, while the SB is fused through a long semirigid linker capable of spanning the distance between the Fc-domain and the antigen-binding site of the antibody (Figure S2b). This linker is similar to the linker used in our LUMABS sensors and consists of 3 flexible domains containing GSG repeats, each separated by 2 α-helical domains consisting of 6 EAAAK repeats.^20,21^ In the absence of the target analyte, the tethered competitor, incorporated in the semirigid linker at the N-terminus of the SB, can bind to the antigen-binding site of the antibody, thereby shifting the equilibrium toward the open conformation of the LUCOSI with an ensuing low bioluminescent signal. In the presence of sufficient target analyte, the competitor can be displaced at the antigen-binding site of the antibody, switching the sensor to a closed conformation with a high bioluminescent signal due to NLuc complementation. The distance between the Fc domain, where Gx is photo-cross-linked, and the antigen-binding site of the antibody ensures that no binding between LB and SB can occur when the sensor is in the open conformation. As a result, the absence of target induces a low luminescent signal. The LUCOS platform thus leverages the architecture of full-size immunoglobulin G (IgG) antibodies. Thermodynamic modeling of the LUCOS system suggested that the response of the sensor could be readily tuned across different analyte concentrations (Figure S2c). Furthermore, these modeling results implied that a competitor with a low nanomolar affinity for the antibody is desired, enabling the efficient open conformation of the sensor in the absence of the target analyte, while allowing competitor displacement in the presence of the target. To enable more robust ratiometric measurements that do not suffer from a decay in absolute signal due to substate depletion, we introduced a green light emitting split calibrator luciferase to the LUCOSI platform (Figure 1c).^5,19,22^ This split calibrator luciferase comprises of a genetic fusion of mNeonGreen (mNG) with split NLuc and allows for in-sample calibration. To this end, the presence of the target analyte instigates a change in emission from green light, emitted by the split calibrator luciferase, to more blue light, due to analyte-induced reconstituted NLuc (Figure 1d).

We also explored a second sensor format that is intrinsically ratiometric and therefore does not require the addition of a separate calibrator luciferase. This second LUCOS platform (LUCOSR) comprises of an additional lower affinity SB2 peptide (*K*_D_ = 190 μM),^9^ fused to the LB domain via a flexible 34 amino acid GGS linker, and the green fluorescent protein mNG, tightly fused to the N-terminus of the higher affinity SB1 (Figure 1a). When the target analyte is absent, the competitor will bind to the antigen-binding site of the antibody. This impedes the effective binding of SB1 to the LB and shifts the equilibrium toward an SB2–LB complex, hence generating a blue luminescent signal. Subsequent displacement of the competitor by the target analyte induces the closed state of LUCOSR due to binding of the higher affinity SB1 to LB. This brings mNG in close proximity to the active site of the reconstituted NLuc, allowing for efficient BRET and the emission of green light. Consequently, the ratio between the emitted green and blue light is a measure for the concentration of the target analyte (Figure 1b).

### Construction and Performance of LUCOS for Small Molecule Detection

To demonstrate the potential of LUCOS for small molecule detection, we developed LUCOSI and LUCOSR sensors for the quantification of cortisol. Cortisol is a steroid hormone that can function as a biomarker for psychological stress and is known to play essential roles in the homeostasis of many systems, including the immune and the cardiovascular system.^23−27^ Cortisol is present in various bodily fluids, including plasma, urine, saliva, and sweat, and its concentrations typically fluctuate throughout the day.^23,28−30^ To this end, the LUCOS-base proteins were expressed in *E. coli* and then purified using Ni^2+^ affinity chromatography followed by Strep–Tactin purification (Figures S12 and S13). SDS-PAGE analysis confirmed the production of pure LUCOS-base proteins and Q-ToF LC-MS demonstrated the successful incorporation of the photo-cross-linkable non-natural amino acid *p*-benzoylphenylalanine in the protein G adapter domain (Figure S3). Subsequently, the sensors were fully assembled in two steps. First, the analyte–competitor was site-specifically incorporated via cysteine–maleimide chemistry to a cysteine residue present at the N-terminus of the SB or mNG domain for LUCOSI or LUCOSR, respectively (step 1 in Figure 2a). Accordingly, we synthesized a cortisol-3 cmo-maleimide from 1-(2-aminoethyl)maleimide and cortisol-3-CMO using HATU coupling. Successful synthesis of the desired product was conformed with LC-MS analysis (Figure S4). Subsequently, we coupled this molecule to the cysteine to serve as an analyte–competitor. Q-ToF LC-MS analysis demonstrated full conversion of LUCOS-base proteins to competitor-labeled sensor proteins (Figures 2b,c and S5). Next, cortisol-functionalized LUCOSR and LUCOSI-base proteins were photo-crosslinked to the constant Fc-domain of a cortisol-specific antibody through covalent protein G coupling under UV-light irradiation (step 2 in Figure 2a). Figure 2d shows the successful photoconjugation of the anticortisol antibody with cortisol-LUCOS-base proteins (1:1 molar ratio), yielding a mixture of nonconjugated, monoconjugated ,and biconjugated antibodies. The reaction mixture was not purified further, as almost no unconjugated cortisol-LUCOS-base protein remained that could contribute to undesired background signal. Separation of the nonconjugated, monoconjugated, and biconjugated antibodies was also deemed unnecessary, as target binding to the free antigen-binding site in the two former species will not significantly influence the sensor’s response range, given the low sensor concentrations that are used.

**Figure 3:**
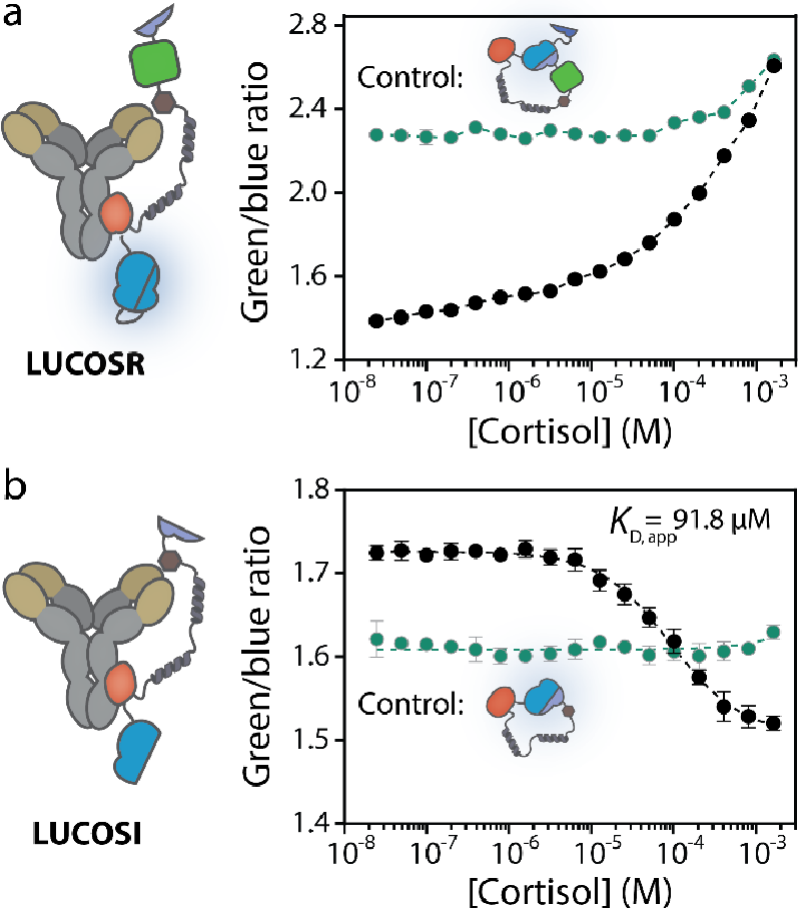
Characterization of the cortisol–LUCOS sensors. (a) The green-to-blue sensor output of the cortisol–LUCOSR sensor (250 pM) in response to increasing cortisol levels. (b) Green-to-blue ratios of the cortisol–LUCOSI (250 pM with 50 pM split calibrator luciferase) in response to different concentrations of cortisol. The titrations were done in PBS buffer (pH 7.4, 0.1% (w/v) BSA, 5% DMSO). The black circles represent the data of the antibody-conjugated LUCOS, and the green circles correspond to the output of the unconjugated control LUCOS-based proteins. Circles represent mean values ± S.D. from technical replicates, with *n* = 3 independent preparations of the analyte.

Subsequent to the successful construction of the LUCOS sensors, we applied the assembled LUCOSR mixture to measure cortisol in PBS buffer (pH 7.4, 0.1% (w/v) BSA, 5% DMSO). To this end, increasing concentrations of cortisol were added to 250 pM of LUCOSR (Figures 3a and S6). In the absence of cortisol, the sensor showed a low green-to-blue ratio, confirming that the sensor is in the open conformation with the cortisol–competitor bound to the antigen-binding site. The subsequent addition of cortisol resulted in an increase in green light, which is consistent with the displacement of the competitor by free cortisol, generating the closed, high BRET state of the sensor (maximal change in the emission ratio = 104% ± 8%). It was not possible to accurately determine the *K*_D,app_ of LUCOSR for free cortisol because the emission ratio did not reach saturation. Please note that a small increase in the emission ratio was also observed with the unconjugated control at high concentrations of cortisol. This effect might be a result of nonspecific binding of cortisol to the protein surface of LUCOSR, generating a conformation that slightly increases BRET efficiency. Next, to evaluate the LUCOS platform’s performance in a more complex medium, we measured cortisol levels in 1:5 diluted human serum. The cortisol–LUCOSR sensor displayed similar performance in both a simple buffer and serum, indicating its potential for POC applications (Figure S7).

After confirming the sensing mechanism with LUCOSR, we next analyzed the performance of the LUCOSI variant. Accordingly, increasing amounts of cortisol were added to 250 pM LUCOSI with 50 pM calibrator luciferase. The sensor exhibited a high green-to-blue ratio at target concentrations below ∼10 μM, suggesting the open state of the sensor with an ensuing low blue light emission (Figure 3b). At higher analyte concentrations, LUCOSI displayed a cortisol–dependent decrease in green-to-blue ratio, consistent with the displacement of the tethered cortisol–competitor by free cortisol from the antigen-binding site of the antibody. In contrast, the unconjugated LUCOSI control did not display a cortisol-dependent change in the emission ratio, indicating that antibody conjugation and the corresponding open state of the sensor are required for target analyte sensing. The *K*_D,app_ of LUCOSI for free cortisol was 92 ± 6 μM, enabling the detection of cortisol in the micromolar concentration range. The anticortisol antibody itself exhibited a higher affinity for free cortisol and free competitor (*K*_D_ = 1.5 nM), as determined by surface plasmon resonance (SPR, Figure S8a). As the affinity of the competitor and the target analyte for the antibody are similar, the sensor will switch to the closed conformation only when the concentration of analyte is approaching the effective local concentration of the competitor, which is likely to be in the 100 μM regime. Taken together, these results show that the LUCOS sensor platform allows the ratiometric detection of micromolar concentrations of the small molecule cortisol through an analyte-induced conformational change in the sensor.

### Performance of LUCOS for Protein Biomarkers

**Figure 4:**
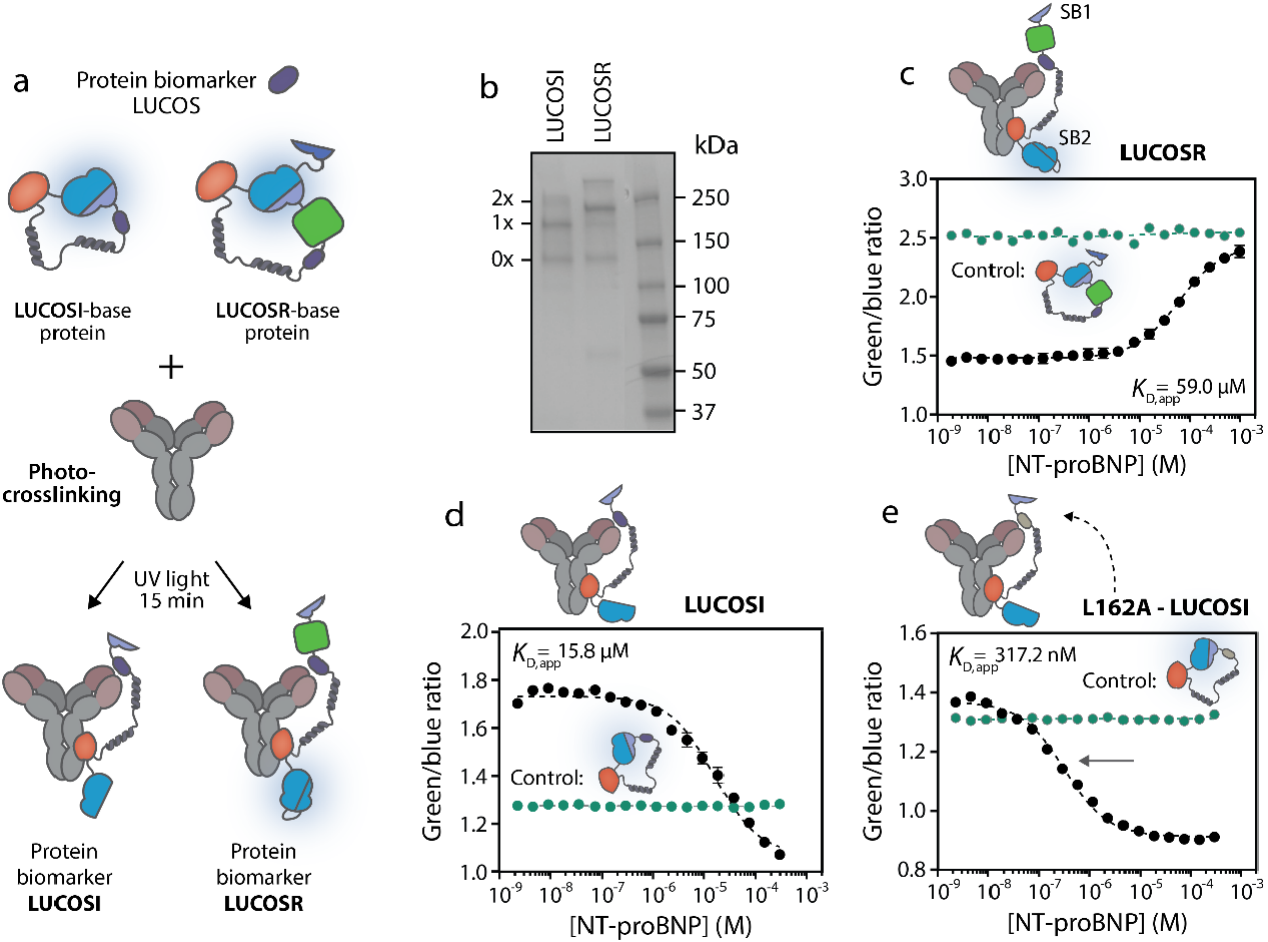
Characterization of the LUCOS sensors for NT-proBNP immunogenic peptide detection. (a) Schematic representation of the development of LUCOSI and LUCOSR for the detection of protein biomarkers. The sensor components contain a genetically incorporated competitor peptide, eliminating the need for labeling with a maleimide–competitor after recombinant expression. The only required step is the photo-cross-linking of the fusion proteins to the analyte-specific antibody. Photo-crosslinking was done in PBS (pH 7.4) for 15 min under ultraviolet (UV) light (λ = 365 nm). (b) Nonreducing SDS-PAGE analysis of the photoconjugation between LUCOS-based proteins and the anti-NT-proBNP antibody, performed in PBS buffer (pH 7.4) in a 1:1 molar ratio. (c) Performance of the NT-proBNP-LUCOSR (100 pM). (d) LUCOSI (250 pM with 50 pM split calibrator luciferase) response curve for increasing concentrations of NT-proBNP immunogenic peptide. (e) Ratiometric sensor output of the mutated L162A-LUCOSI (250 pM) with 50 pM split calibrator luciferase. All titrations were performed in PBS buffer (pH 7.4, 0.1% (w/v) BSA). The black curve represents the data of the antibody-conjugated LUCOS and the green curves correspond to the green-to-blue ratios of the unconjugated control LUCOS. Circles represent mean values ± S.D. from technical replicates, with *n* = 3 independent preparations of the analyte (except for the LUCOSR control which is *n* = 1).

Next, to demonstrate the potential of LUCOS for the detection of protein biomarkers, we developed sensors for the quantification of the N-terminal prohormone of B-type natriuretic peptide (NT-proBNP), a popular biomarker to identify patients with heart failure that is secreted from cardiomyocytes in response to mechanical stretching.^31−38^ To develop NT-proBNP LUCOS sensors, we first genetically incorporated the antibody-binding epitope part of the NT-proBNP peptide in LUCOSI and LUCOSR, and subsequently successfully expressed these fusion proteins in *E. coli* (Figures4a, S9, S14, and S15). Next, the LUCOS-based proteins were photo-crosslinked to an anti-NT-proBNP antibody, generating a sensor mixture of nonconjugated, once-conjugated, and twice-conjugated antibodies (Figure 4b). This sensor mixture was subsequently applied to measure increasing concentrations of the immunogenic peptide of NT-proBNP (Figure S14). Figure 4c shows that LUCOSR adopts an open conformation at NT-proBNP immunogenic peptide concentrations below ∼5 μM due to the relatively high affinity of the peptide for the antibody (*K*_D_ = 6.9 nM, Figure S8b). At higher analyte concentrations, the tethered competitor can be competed from the antigen-binding site by free NT-proBNP immunogenic peptide, and the sensor adopts the closed conformation, with an increased BRET efficiency (maximal change in emission ratio = 72% ± 3%, Figure S10a). As expected, the unconjugated control did not display this analyte-dependent increase in green-to-blue ratio. In an effort to further increase the maximal change in the emission ratio of LUCOSR, we also tested a LUCOSR variant with increased SB1 and SB2 affinities (*K*_D_ = 180 nM and *K*_D_ = 2.5 μM, respectively, Figure S13).^9,39^ However, this sensor yielded a relatively high green-to-blue ratio in the absence of the target analyte and did not display an increase in the emission ratio upon addition of varying concentrations of NT-proBNP immunogenic peptide (Figure S11). This is unexpected as the increase in affinity of SB2 was larger than that of SB1 and is possibly due to intermolecular complex formation between two LUCOSR sensors. Irrespective, these results show that our initial choice of SB affinities (SB1, *K*_D_ = 2.5 μM and SB2, *K*_D_ = 190 μM) already yielded optimal changes in the emission ratio.

A decrease in the emission ratio was observed upon addition of increasing concentrations of the NT-proBNP immunogenic peptide to the NT-proBNP LUCOSI sensor (maximal change = 74% ± 7%), which is consistent with the displacement of the competitor at high analyte concentrations (Figures 4d and S10b). The LUCOSI exhibited a higher apparent affinity for free NT-proBNP immunogenic peptide compared to LUCOSR (*K*_D,app_ = 16 ± 2 μM versus 59 ± 3 μM, respectively). This difference is probably due to the absence of an additional SB2 domain in LUCOSI, shifting the dose–response curve of this sensor to lower NT-proBNP immunogenic peptide concentrations. In an effort to further increase the affinity of the LUCOSI sensor for the free NT-proBNP immunogenic peptide, we aimed to attenuate the affinity of the competitor for the antibody, while still favoring the open conformation of the sensor in the absence of the target. Accordingly, we mutated a hydrophobic leucine residue in the competitor peptide to an alanine (L162A, Figures S14 and S15). Similar to the previous LUCOS sensors for NT-proBNP immunogenic peptide detection, the L162A-LUCOSI variant was expressed in *E. coli* and purified using Ni^2+^ affinity chromatography followed by Strep–Tactin purification. The mutation indeed increased the apparent affinity of the sensor for free target approximately 50-fold (*K*_D,app_= 317 ± 26 nM), allowing the detection of the NT-proBNP immunogenic peptide in the nanomolar concentration range (Figures 4e and S10c). The change in the emission ratio of 57 ± 1% was slightly lower than that of the original LUCOSI sensor, which is probably due to some of the sensor already being in the closed state even in the absence of analyte (see also Figure S2c). Collectively, these results illustrate that the LUCOS sensors enable the detection of protein biomarkers and that the detectable concentration range can be readily tuned by adapting the affinity of the tethered competitor.

## Conclusions

In this work, we established LUCOS as a competition-based bioluminescent sensor platform and applied it for the detection of both a small molecule and a protein biomarker target (cortisol and NT-proBNP, respectively). The employment of full-sized antibodies makes the LUCOS platform modular by design because it eliminates the need for genetic incorporation of an analyte-binding domain and omits necessary geometry optimalization to achieve high ratiometric responses.^15^ Furthermore, the LUCOS platform exploits general sensor base proteins that can easily be tailored for the detection of new targets by simple exchange of the competitor. The cysteine residue in the small molecule LUCOS allows straightforward incorporation of a new competitor by maleimide-based conjugation chemistry, while the development of protein biomarker LUCOS sensors is achieved by simple exchange of the antibody-binding competitor sequence. The usage of Gx adapter-based photo-crosslinking enables efficient and easy conjugation of these LUCOS-base proteins to different antibodies and consequently should allow the detection of a wide variety of relevant analytes.

To enable analyte detection in the relevant concentration ranges, the sensitivity of the herein developed LUCOS sensors should be further improved.^31,33,40,41^ However, we have demonstrated that by attenuating the binding affinity of the competitor in the protein biomarker LUCOS, the measurable range shifts to lower analyte concentrations. This suggests that the response of the LUCOS sensors can be tuned to clinically relevant concentrations by increasing or decreasing the affinity of the tethered competitor, enabling the detection of the target above or below the effective molarity. For peptide epitopes, such as the NT-proBNP competitor, mutations could be introduced to attenuate the affinity for the antibody, while analyte analogues could be used in the small molecule LUCOS. However, it is important to ensure that the affinity of the competitor remains sufficiently high to favor the open state of the sensor in the absence of target. Hence, the LUCOS sensors are suitable for the detection of analytes in the nanomolar concentration regime or higher (Figure S2). Alternatively, optimization of the stiffness of the linker between the Gx adapter and the high affinity SB1 might affect the effective molarity of the competitor and influence the equilibrium between the closed and the open states of the sensor.^42^ We believe that the use of peptide-based competitors will be most attractive in future designs, in particular for analytes for which competing ligands are unknown. First, peptide-based competitors can be genetically introduced, avoiding the need for a post-translational conjugation step. Second, various display technologies, such as phage and yeast, display can be used to screen for peptide-based inhibitors binding at the antigen-binding site, using competitive elution with the analogue during selection. However, the most promising future approach will be to harness the rapidly increasing power of deep learning to computationally design peptide-based competitors that can be experimentally tested directly in the context of the LUCOS sensor.^43,44^

Overall, the modular design of the LUCOS platform allows for easy optimization and screening of different affinity competitors, SB variants, and linker stiffness to obtain sensors that enable measurements in the relevant concentration range and simultaneously yield high maximal changes in the emission ratio. The LUCOS platform complements RAPPID by enabling the modular detection of small molecules in the nanomolar-to-micromolar concentration range. Furthermore, the intrinsic ratiometric and bioluminescent readout of the LUCOS sensors render the platform a promising addition to the POC sensor toolbox. To realize this, we envision the integration of LUCOS sensors into easy-to-use POC devices, enabling the detection of a broad range of relevant small molecules and protein biomarkers outside of clinical laboratories.

## Data Availability

Plasmids and DNA sequences are available via Addgene.

## Supporting Information Available

Thermodynamic model of LUCOSI, SDS-PAGE analysis of protein expression and purification, synthesis of cortisol–competitor, ESI-QTOF chromatogram of competitor-coupled LUCOS, performance of LUCOS in diluted blood serum, SPR analysis, performance of LUCOS SB variant, and DNA and amino acid sequences of the different LUCOS variants (PDF)
