# Supplementary material for: Turning Antibodies into Ratiometric Bioluminescent Sensors for Competition-Based Homogeneous Immunoassays
Source: ACS Sens. 2024 Feb 21;9(3):1401–9. doi: 10.1021/acssensors.3c02478 (PMC10964239; doi:10.1021/acssensors.3c02478)
Supplement: Supplementary file 1 — se3c02478_si_001.pdf [file se3c02478_si_001.pdf]

Turning antibodies into ratiometric bioluminescent sensors for competition-based homogenous immunoassays

Eva A. van Aalen[ab], Joep J. J. Lurvink[ab], Leandra Vermeulen[ab], Benice van Gerven[ab], Yan Ni[ab], Remco Arts[ab] and Maarten Merkx*[ab]

[a] Laboratory of Chemical Biology, Department of Biomedical Engineering, Eindhoven University of Technology, P.O Box 513, 5600 MB Eindhoven, The Netherlands.

[b] Institute for Complex Molecular Systems, Eindhoven University of Technology, P.O Box 513, 5600 MB Eindhoven, The Netherlands.

*Corresponding author: Maarten Merkx, m.merkx@tue.nl.

Figure S1	Thermodynamic scheme describing the interactions in the LUCOSI assay	S2
Figure S2	Simulation of LUCOSI response curve for different parameters	S5
Figure S3	Reducing SDS-PAGE analysis of LUCOS-base proteins	S6
Figure S4	Synthesis, purification, and characterization of cortisol-3-CMO-malemide	S7
Figure S5	ESI-QTOF chromatogram of LUCOS coupled to cortisol-3-CMO-malemide	S8
Figure S6	Luminescent spectra of the LUCOS sensors for cortisol sensing	S8
Figure S7	Dose-response curve of cortisol-LUCOSR in 20% human blood serum	S9
Figure S8	Binding affinities of NLuc-cortisol-3-CMO-malemide and NT-proBNP	S9
Figure S9	Nonreducing SDS-PAGE analysis of LUCOS-base proteins with NT-proBNP competitor	S10
Figure S10	Luminescent spectra of the LUCOS sensors for NT-proBNP immunogenic peptide sensing	S10
Figure S11	Performance of LUCOSR variant with alternative SB affinities	S11
Figure S12	Amino acid sequence of the basic LUCOSI	S11
Figure S13	Amino acid sequence of the basic LUCOSR	S11
Figure S14	Amino acid sequence of the NT-proBNP immunogenic peptide for LUCOSI	S12
Figure S15	Amino acid sequence of the NT-proBNP immunogenic peptide for LUCOSR	S12
	Supporting references	S12

Thermodynamic model

To gain a better insight into the response of the LUCOS sensors to the presence of the target analyte, we developed a thermodynamic model that defines the equilibrium equations involved in analyte-induced switching of the sensors. First, we described the LUCOSI system by identifying all unique binding states between sensor and analyte and described the interactions between the unique binding states with reversible reactions. Figure S1 schematically displays the unique binding states and their interactions for the single-conjugated LUCOSI sensor. We consider a reaction scheme where a sensor molecule (S) can bind two analyte molecules (C) simultaneously. In the absence of analyte, the analyte analog is expected to be bound to the paratope region of the antibody (S_{open}). In the presence of target, the sensor can adopt a conformation with a reconstituted NLuc (S_b) after dissociation of the analyte analog from the paratope region. Analyte binding to the antibody (S_bC & SC) prevents rebinding of the analyte analog, thereby shifting the equilibrium of the sensor towards the closed conformation. Now, only one of the antibody paratope regions is accessible to the analyte analog. The second antibody paratope region can bind analyte (C_r) but does not affect the sensor mechanism.

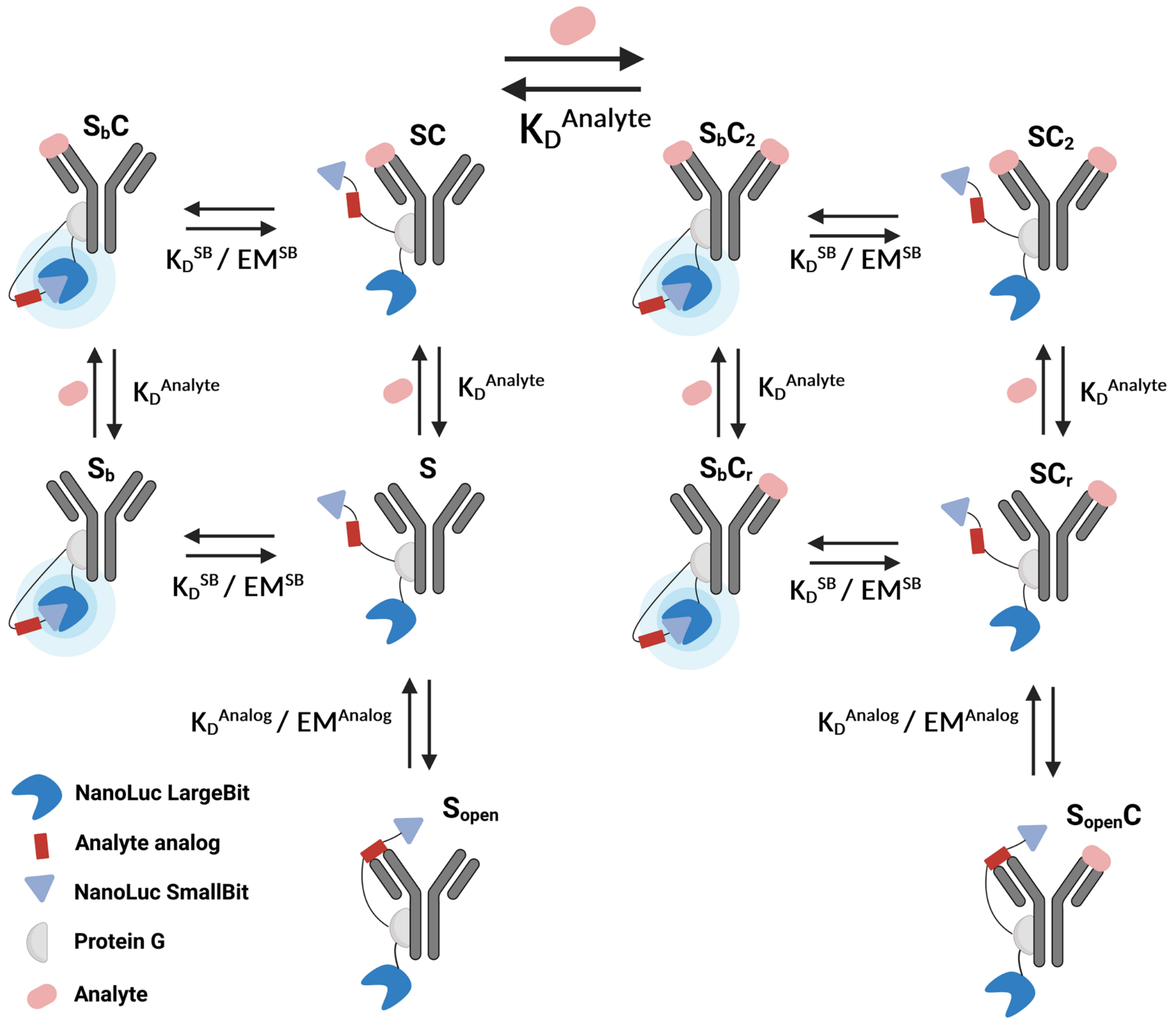

Figure S1 | Thermodynamic scheme describing the complexes and conformational states of the LUCOSI sensor.

Although intermolecular binding between adapter proteins is possible, the concentration of such a complex is assumed low enough to be omitted from the model. In addition, the presence of non-conjugated antibodies and adapter proteins is not accounted for. Furthermore, to keep the number of possible sensor states manageable we only considered monoconjugates and restricted the analyte analog to bind only to

the closest antigen binding site. These two assumptions will have only minor effects on the outcome of the model (resulting from statistical effects of max a factor 2), and thus still allow insight into the relative importance of the various effective concentrations and the affinities of the antibody and split luciferase interactions.

$[C]_{tot}$ = Total concentration of analyte
$[S]_{tot}$ = Total concentration of sensor

$K_D^{Analyte}$ = dissociation equilibrium constant for the binding of analyte to the antibody
K_D^{Analog} = dissociation equilibrium constant for the binding of analyte analog to the antibody
K_D^{SB}= dissociation equilibrium constant for the binding of small BiT to large BiT

EM^{Analog}= Effective molarity of analog binding of the antibody
EM^{SB}= Effective molarity of small BiT binding to large BiT

[SC] = Free concentration of analyte-bound sensor in catalytically inactive state
$[SC_r]$ = Free concentration of right-side analyte-bound sensor in catalytically inactive state
$[SC_2]$ = Free concentration of double analyte-bound sensor in catalytically inactive state

$[S_b]$ = Free concentration of sensor with reconstituted NLuc
$[S_bC]$ = Free concentration of analyte-bound sensor with reconstituted NLuc
$[S_bC_r]$ = Free concentration of right-side analyte-bound sensor with reconstituted NLuc
$[S_bC_2]$ = Free concentration of double analyte-bound sensor with reconstituted NLuc

$[S_{open}]$ = Free concentration of analog-bound sensor in catalytically inactive state
$[S_{open}C]$ = Free concentration of (right side analyte + left side analog)-bound sensor in catalytically inactive state

The model was implemented using a general framework for equilibrium models developed by Geertjens *et al.*[1], using the following input to build the model in the Python tool:

Input:	Python script:
$K_D^{Analyte} = [S] * [C] / [SC]$	S + C = SC; Kdc
$K_D^{Analyte} = [S_b] * [C] / [S_bC]$	Sb + C = SbC; Kdc
$K_D^{Analyte} = [SC_r] * [C] / [SC_2]$	SCr + C = SCrC; Kdc
$K_D^{Analyte} = [S_bC_r] * [C] / [S_bC_2]$	SbCr + C = SbCrC; Kdc
$K_D^{analog} / EM^{analog} = [S] / [S_{open}]$	SCr = SopenCr; Kda / Ema
$K_D^{analog} / EM^{analog} = [SC_r] / [S_{open}C]$	S = Sopen; Kda / Ema
$K_D^{SB} / EM^{SB} = [SC] / [S_bC]$	SC = SbC; Kdsb / Emsb
$K_D^{SB} / EM^{SB} = [S] / [S_b]$	S = Sb; Kdsb / Emsb
$K_D^{SB} / EM^{SB} = [SC_2] / [S_bC_2]$	SCrC = SbCrC; Kdsb / Emsb
$K_D^{SB} / EM^{SB} = [SC_r] / [S_bC_r]$	SCr = SbCr; Kdsb / Emsb
$K_D^{Analyte} = [S_{open}] * [C] / [S_{open}C]$	Sopen + C = SopenCr; Kdc
$K_D^{Analyte} = [S_b] * [C] / [S_bC_r]$	Sb + C = SbCr; Kdc
$K_D^{Analyte} = [S] * [C] / [SC_r]$	S + C = SCr; Kdc
$K_D^{Analyte} = [S_bC] * [C] / [S_bC_2]$	SbC + C = SbCrC; Kdc
$K_D^{Analyte} = [SC] * [C] / [SC_2]$	SC + C = SCrC; Kdc

From this, a comprehensive model was generated within the framework. A detailed model is presented below.

The concentrations of the dependent species at equilibrium can be determined utilizing the concentrations of the independent species and the corresponding equilibrium constant, using the following relations:

CS= C*S/Kdc

CSb= C*Emsb*S/(Kdc*Kdsb)
CCrS= C**2*S/Kdc**2
CCrSb= C**2*Emsb*S/(Kdc**2*Kdsb)
CrSopen= C*Ema*S/(Kda*Kdc)
Sopen= Ema*S/Kda
Sb= Emsb*S/Kdsb
CrSb= C*Emsb*S/(Kdc*Kdsb)

CrS= C*S/Kdc

The mass balance of the independent species in terms of free and complexed forms:

C_tot= C + 2*CCrS + 2*CCrSb + CS + CSb + CrS + CrSb + CrSopen
S_tot= CCrS + CCrSb + CS + CSb + CrS + CrSb + CrSopen + S + Sb + Sopen

Substituting the relations above in the mass balance equations gives:

C_tot = 2*C**2*Emsb*S/(Kdc**2*Kdsb) + 2*C**2*S/Kdc**2 + C*Ema*S/(Kda*Kdc) + 2*C*Emsb*S/(Kdc*Kdsb) + C + 2*C*S/Kdc

S_tot = C**2*Emsb*S/(Kdc**2*Kdsb) + C**2*S/Kdc**2 + C*Ema*S/(Kda*Kdc) + 2*C*Emsb*S/(Kdc*Kdsb) + 2*C*S/Kdc + Ema*S/Kda + Emsb*S/Kdsb + S

Lastly, these equations were rewritten to equal zero, and subsequently divided by the total concentrations on both sides. We applied this approach to expedite the solving process and reach a solution more efficiently:

C = (2*C**2*Emsb*S/(Kdc**2*Kdsb) + 2*C**2*S/Kdc**2 + C*Ema*S/(Kda*Kdc) + 2*C*Emsb*S/(Kdc*Kdsb) + C + 2*C*S/Kdc - C_tot) / C_tot = 0

S = (C**2*Emsb*S/(Kdc**2*Kdsb) + C**2*S/Kdc**2 + C*Ema*S/(Kda*Kdc) + 2*C*Emsb*S/(Kdc*Kdsb) + 2*C*S/Kdc + Ema*S/Kda + Emsb*S/Kdsb + S - S_tot) / S_tot = 0

The LUCOS sensor is a one-component sensor that changes its conformation based on intramolecular binding events. Intramolecular tethering of binding partners results in an increase in local concentration thereby enhancing the binding affinity.[2] The increased local concentration can be expressed as effective molarity (EM) and is defined by the ratio between the equilibrium constant of the intramolecular reaction and the equilibrium constant of the corresponding intermolecular reaction. The properties of the linker (length, stiffness) and the distance that the linker bridges in the complex determine the EM.[2] In order to create an accurate model, the equations established by the system description need to account for the EM when they describe an intramolecular binding event. The LUCOS platform contains a variety of linkers in between intramolecular binding partners (analyte analog to antibody, SB to LB). The effect of these linkers on the EM between binding partners was modelled using the C_{eff} calculator developed by Kjaergaard *et al.*[3] Using PyMOL, distances between linker termini (protein G to paratope region and SB to LB) were determined, resulting in estimates of 8.5 nm and 2 nm, respectively. The C_{eff} calculator is developed for homopolymer disordered linkers such as GGS linkers. However, the semi-rigid linker in LUCOS contains alpha helical forming domains, which will result in a higher effective persistence length than fully flexible linkers. Therefore, the C_{eff} calculator input values need to be adjusted to account for the effect of the semi-rigid linker. In our modelling we chose a persistence length of 7.8 Å as a baseline parameter, which yields values of EM_{Analog} = 191 µM and $EM_{SmallBit}$ = 1.08 mM). However, we also modelled the effect of using lower persistence lengths (4.5 Å ; fully flexible SSG linker) and much more stiffer linkers (persistence lengths of 18 and 36 Å).

To illustrate the dependence of the LUCOSI sensor response on different parameters, simulations were performed that varied analogue binding affinity, analyte binding affinity, small BiT binding affinity and the stiffness of the semi-rigid linker (Figure S2). Figure S2a shows that the use of a higher affinity small BiT generates a relatively more sensitive sensor. However, a strong small BiT binding affinity also yields a higher background signal in the absence of analyte. In contrast to experimental findings, the use of a K_D = 180 nM small BiT was expected to result in a sensitive sensor with low background signal. The difference between model simulations and experimental findings (Figure S8) was contributed to intermolecular binding of small BiT to large BiT, significantly increasing background signal. Furthermore, Figure S2b shows that a more flexible linker between the analogue and protein G adapter is predicted to result in an increase in sensitivity, while more stiffer linkers would actually decrease the apparent affinity of the sensor for its analyte. Although the use of a more flexible linker would be expected to shift the equilibria to the closed sensor conformation, this does not result in a significant increase in background signal. Figure S2c shows that the use of a weaker binding analyte analog shifts the response curve to lower analyte concentrations. However, in these simulations, the background signal in the absence of analyte is significantly increased when a lower affinity analog is used because more sensor is expected to be in the closed conformation with a reconstituted NLuc. Therefore, the simulations suggest that a K_D <50 nM for the analyte analog is required for the sensor to maintain an open conformation in the absence of analyte. This is in line with our experimental findings of the NT-proBNP L162A mutant sensor, where a lower analog affinity resulted in a decreased dynamic range. Finally, attenuating the binding affinity of both the analyte analog and the analyte does not increase the sensitivity of the sensor, since there is no relative change in preference for analyte or analyte analog binding (Figure S2d).

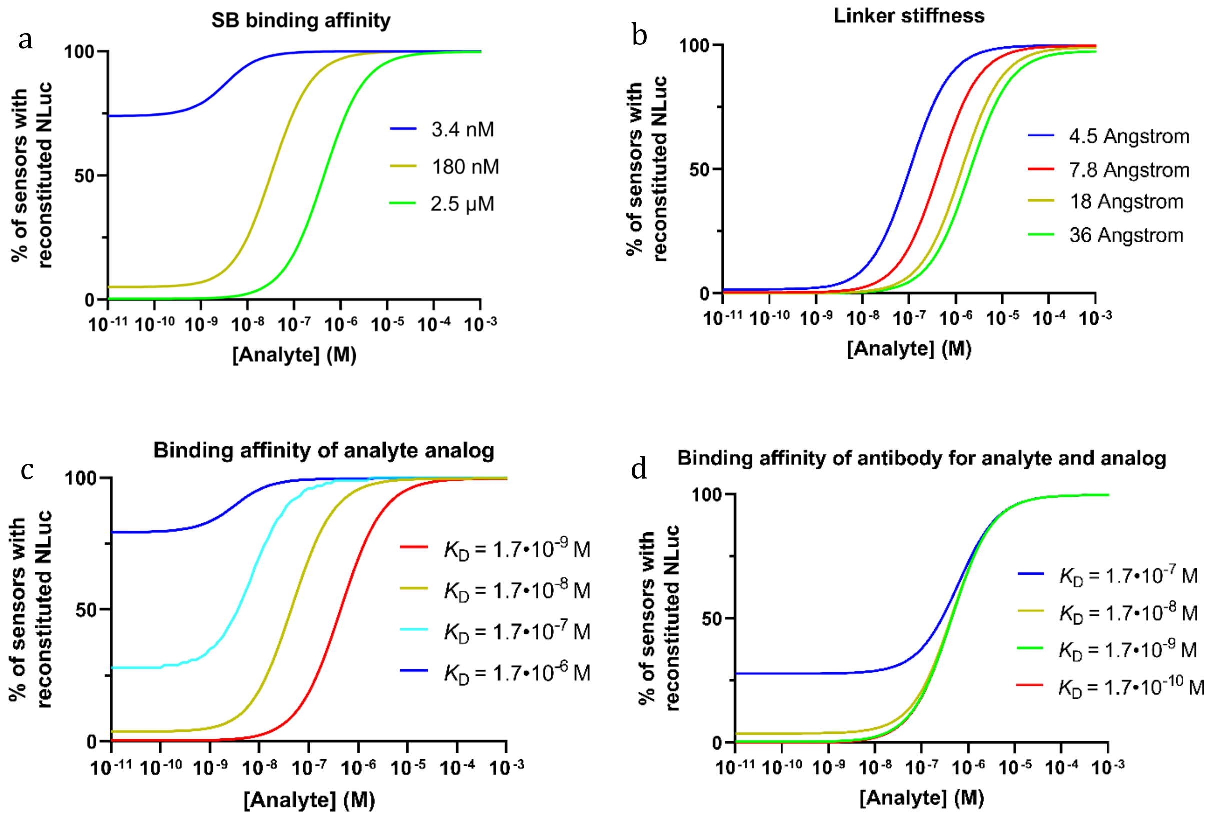

Figure S2 | Simulation of LUCOSI response curve for different parameters. (a) The response curves for different small BiT binding affinities. (b) The response curves with different semiflexible linker stiffness, resulting in a change of EM. (c) The response curves for different analyte analog binding affinities. (d) The response curves for different antibody binding affinities. Baseline parameters: $[S_{tot}]$ = 1 nM, $K_{D,Analog}$ = 1.7 nM, $K_{D,Analyte}$ = 1.7 nM, $K_{D,SB}$ = 2.5 µM, Persistence length semiflexible linker = 7.8 Angstrom, yielding values of EM_{Analog} = 191 µM and $EM_{SmallBit}$ = 1.08 mM.

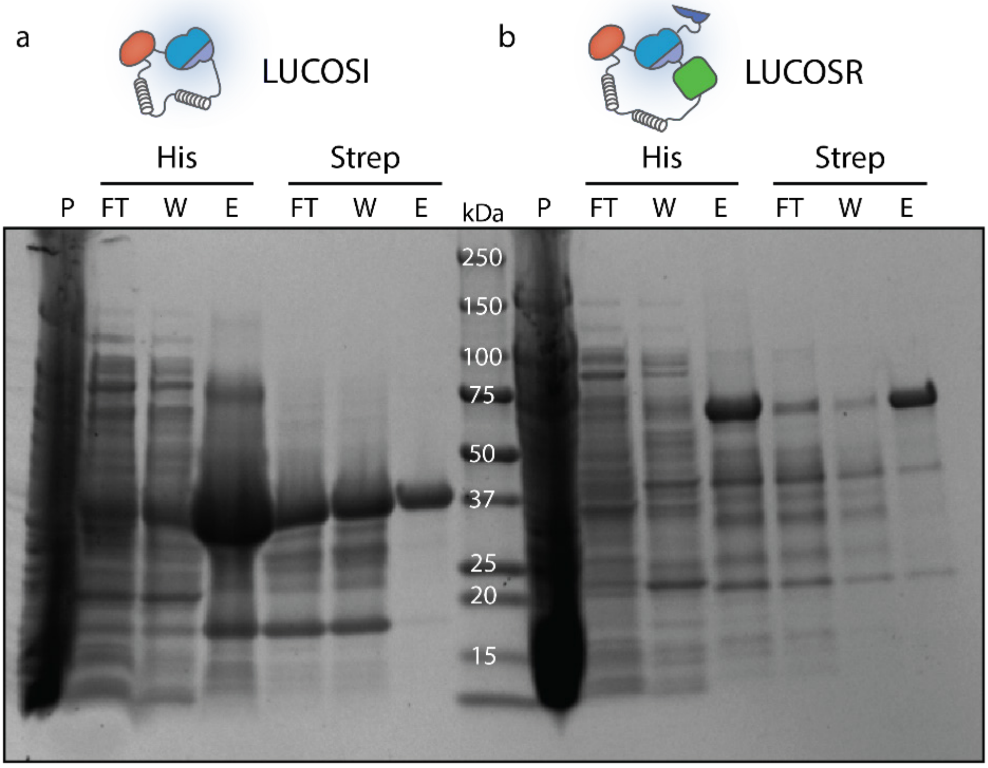

Figure S3 | Reducing SDS-PAGE analysis of LUCOS-base proteins. The *E. coli* expressed (a) LUCOSI (expected mass: 40063 Da) and (b) LUCOSR (expected mass: 69050 Da) proteins were purified using Ni^{2+} affinity chromatography (His) and Strep-Tactin chromatography (Strep). P: pellet, FT: flow through, W: wash, E: elution.

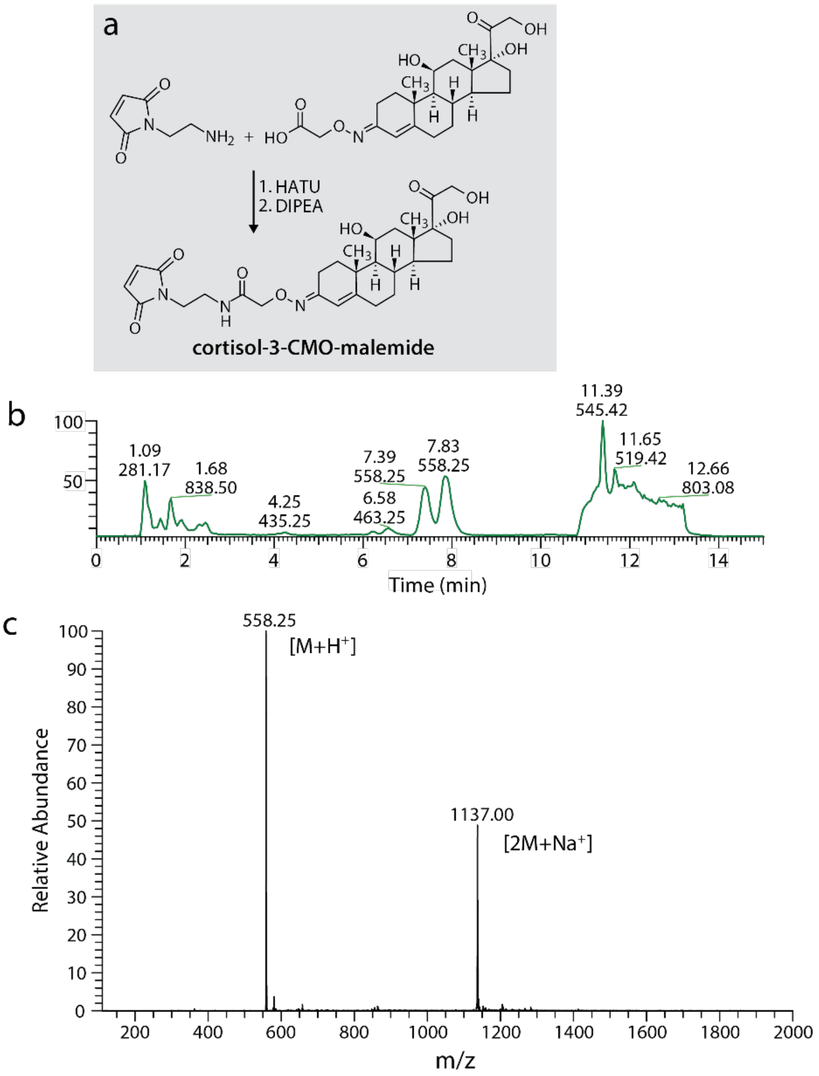

Figure S4 | Synthesis, purification and characterization of cortisol-3-CMO-malemide. (a) Synthesis of cortisol-3-CMO-malemide from 1-(2-aminoethyl)maleimide and cortisol-3-CMO using HATU coupling. (b) Total ion chromatogram of reaction product isocratic elution from a C18 column. The two peaks at 7.39 and 7.83 minutes displayed the expected mass and were collected. (c) ITMS m/z spectrum of the collected fractions, including the expected mass of the product.

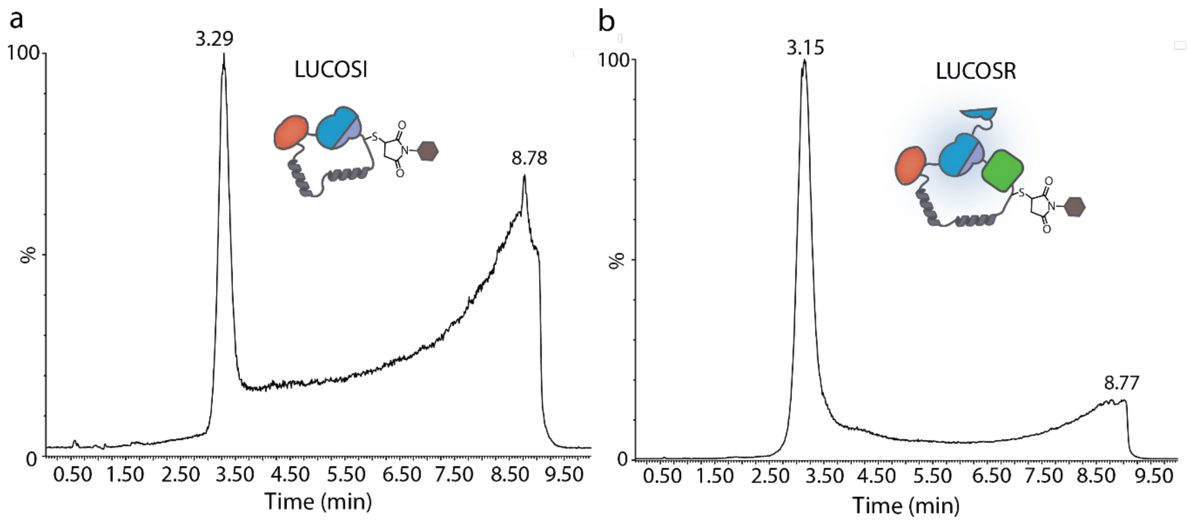

Figure S5 | ESI-QToF chromatogram of LUCOS coupled to cortisol-3-CMO-malemide. (a) LUCOSI (calculated mass without the N-terminal methionine: 40620.6 Da). (b) LUCOSR (calculated mass without the N-terminal methionine: 69607.1 Da). Massa spectra of the chromatograms can be found in Figures 2b and 2c.

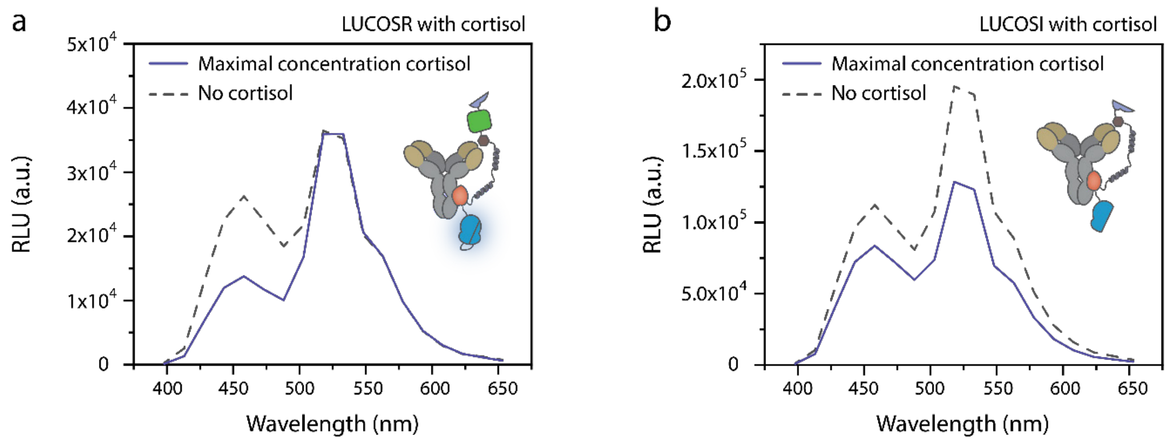

Figure S6 | Luminescent spectra of the LUCOS sensors for cortisol sensing. Luminescent output of (a) LUCOSR (250 pM) and (b) LUCOSI (250 pM; with 50 pM split calibrator luciferase) without cortisol (dotted grey line) or with the maximal concentration of cortisol (solid blue line) displayed in Figure 3a and 3b, respectively. Lines represent the mean of n = 3 and measurements were done in PBS buffer (pH 7.4, 0.1% (w/v) BSA, 5% DMSO).

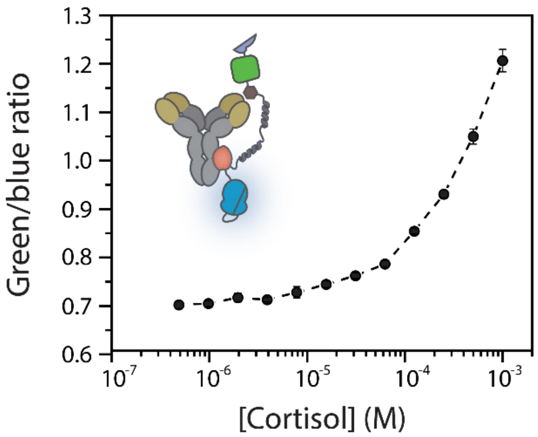

Figure S7 | Dose-response curve of cortisol-LUCOSR in 20% human blood serum. The green-to-blue sensor output of 250 pM LUCOSR in response to increasing cortisol levels. Circles represent mean values ± s.d. from technical replicates and measurements were done in PBS buffer (pH 7.4, 0.1% (w/v) BSA, 5% DMSO) with 20% human serum.

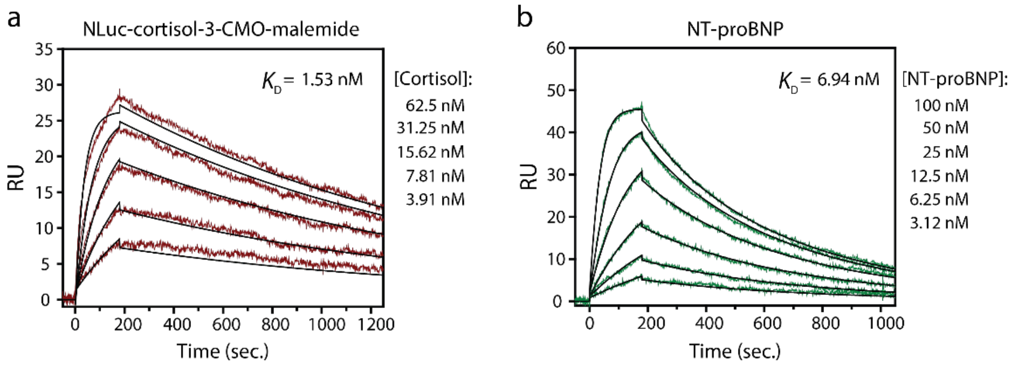

Figure S8 | Determination of binding affinities of NLuc-cortisol-3-CMO-malemide and NT-proBNP using SPR. (a) Surface plasmon resonance (SPR) sensorgram of NLuc-cortisol-3-CMO-malemide with antibody. A cysteine containing NLuc variant was used to covalently couple cortisol-3-CMO-malemide via maleimide chemistry. Subsequently, anti-cortisol antibodies were immobilized on CMD 500M chips (Xantec) and different concentrations of NLuc-cortisol-3-CMO-malemide were flown over the surface in HBS-EP buffer (10 mM HEPES, 150 mM NaCl, 3 mM EDTA, 0.05% v/v P20, pH7.4). (b) SPR sensorgram of the NT-proBNP peptide with antibody. Anti-NT-proBNP antibodies were immobilized on CMD 500M chips and varying concentrations of NT-proBNP were added in HBS-EP buffer. SPR sensorgrams are shown in red and green, fitted curves in black. SPR measurements were executed on a Biacore X100.

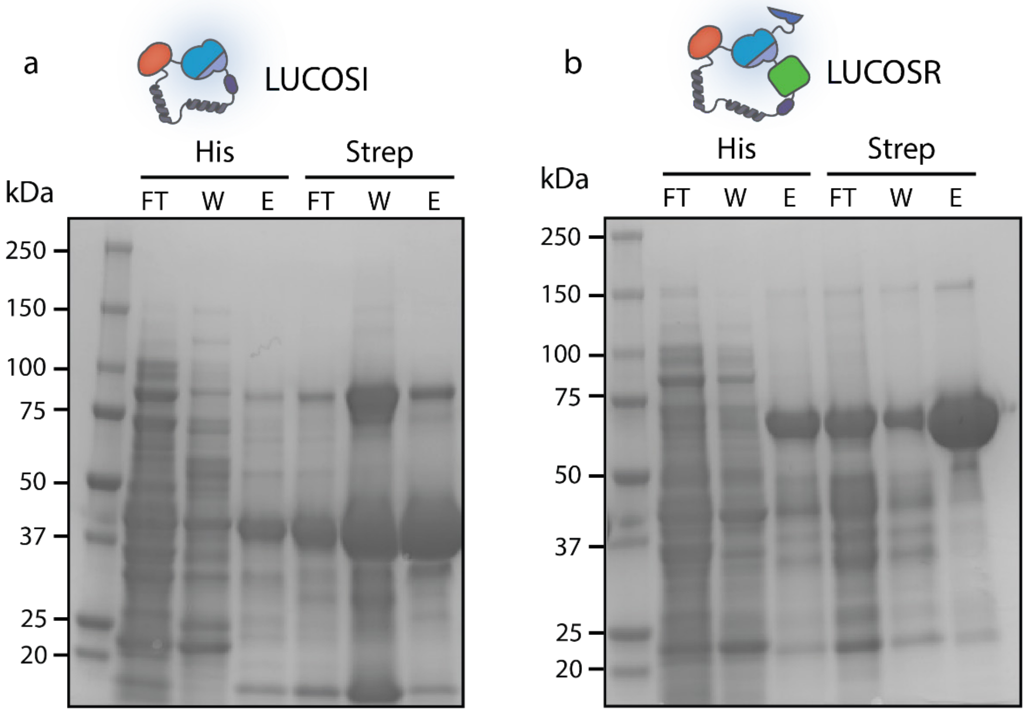

Figure S9 | Nonreducing SDS-PAGE analysis of LUCOS-base proteins with NT-proBNP competitor. (a) LUCOSI (expected mass: 41656 Da) and (b) LUCOSR (expected mass: 70452 Da) sensor components, containing the NT-proBNP competitor peptide, were expressed in *E. coli* and subsequently purified using Ni^{2+} affinity chromatography (His) and Strep-Tactin chromatography (Strep). FT: flow through, W: wash, E: elution.

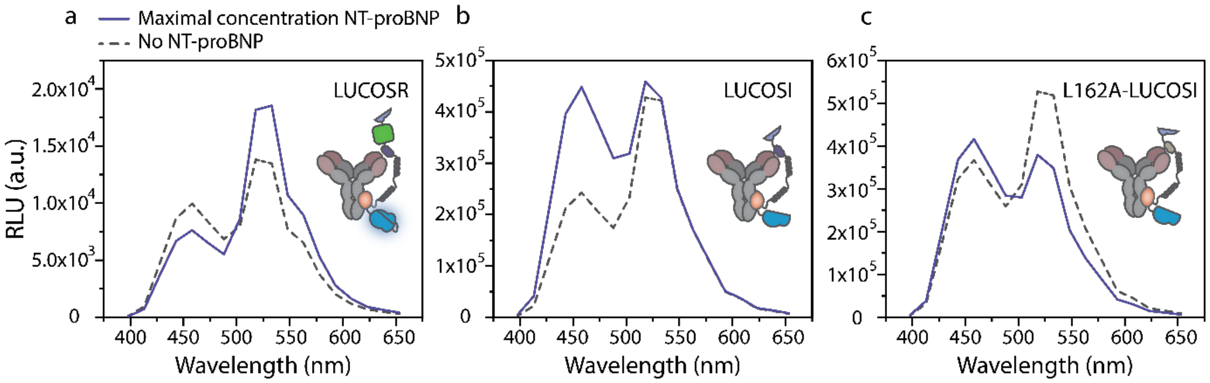

Figure S10 | Luminescent spectra of the LUCOS sensors for NT-proBNP immunogenic peptide sensing. Luminescent output of (a) LUCOSR (100 pM), (b) LUCOSI (250 pM with 50 pM split calibrator luciferase) and (c) L162A-LUCOSI (250 pM with 50 pM split calibrator luciferase) without NT-proBNP immunogenic peptide (dotted grey line) or with the maximal concentration of NT-proBNP immunogenic peptide (solid blue line) displayed in Figure 4c, 4d and 4e, respectively. Lines represent the mean of n = 3 and measurements were done in PBS buffer (pH 7.4, 0.1% (w/v) BSA).

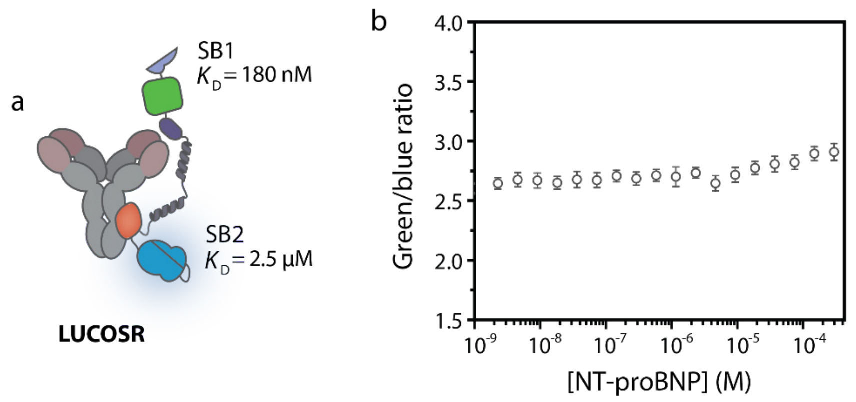

Figure S11 | Performance of LUCOSR variant with alternative SB affinities. (a) Schematic illustration of the LUCOSR variant. The K_D of the higher affinity SB1 was mutated from 2.5 µM to 180 nM and the lower affinity SB2 was mutated from K_D=190 µM to K_D=2.5 µM. (b) Sensor response curve using 100 pM of LUCOSR sensor and increasing concentrations of NT-proBNP in PBS buffer (pH 7.4, 0.1% (w/v) BSA). Circles represent mean values ± s.d. from technical replicates, with *n* = 3 independent preparations of the analyte.

MGSSHHHHHHSSGLVPRGSHMGGSVFTLEDFVGDWEQTAAYNLDQVLEQGGVSSLLQNLAVSVTPIQRIVRSGENALKIDI
HVIIPYEGLSADQMAQIEEVFKVVYPVDDHHFKVILPYGTLVIDGVTPNMLNYFGRPYEGIAVFDGKKITVTGTLWNGNKIIDE
RLITPDGSMLFRVTINSGGSGGSMTFKLIINGKTLKGEITIEAVDA*EAEKIFKQYANDYGIDGEWTYDDATKTFTVTEGTGGSG
GSGGSGGSGGSGGSGAEAAAKEAAAKEAAAKEAAAKEAAAKEAAAKAGSGGSGGSGGSGGSGGSGAEAAAKEAAAKEAAAKE
AAAKEAAAKEAAAKAGSGGSGGSGGSGGSGGSGG**C**TSGGTGSVTGYRLFEKESGGSGGSWSHPQFEK*

Figure S12 | Amino acid sequence of the LUCOSI-base protein. His-tag (pink), LB (cyan), protein G domain (red), amber stop codon (yellow), the restriction sites SpeI and AgeI for NT-proBNP peptide incorporation (teal and dark red, respectively), SB (dark blue) and Strep-tag (gray). The cysteine used for maleimide coupling is shown in bold and underlined.

MGSSHHHHHHSSGLVPRGSHMVTGYRLFEEILGSGGSGGSGGSGGSGSGGSGGSGGSGGSGGSVFTLEDFVGDWEQTAA
YNLDQVLEQGGVSSLLQNLAVSVTPIQRIVRSGENALKIDIHVIIPYEGLSADQMAQIEEVFKVVYPVDDHHFKVILPYGTLVID
GVTPNMLNYFGRPYEGIAVFDGKKITVTGTLWNGNKIIDERLITPDGSMLFRVTINSGGSGGSMTFKLIINGKTLKGEITIEAVD
A*EAEKIFKQYANDYGIDGEWTYDDATKTFTVTEGTGGSGGSGGSGGSGGSGGSGAEAAAKEAAAKEAAAKEAAAKEAAAKE
AAAKAGSGGSGGSGGSGGSGGSGAEAAAKEAAAKEAAAKEAAAKEAAAKEAAAKAGSGGSGGSGGSGGSGGASSGG**C**TSGH
MVSKGEEDNMASLPATHELHIFGSINGVDFDMVGQGTGNPNDGYEELNLKSTKGDLQFSPWILVPHIGYGFHQYLPYPDGM
SPFQAAMVDGSGYQVHRTMQFEDGASLTVNYRYTYEGSHIKGEAQVKGTGFPADGPVMTNSLTAADWCRSKKTYPNDKTII
STFKWSYTTGNGKRYRSTARTTYTFAKPMAANYLKNQPMYVFRKTELKHSKTELNFKEWQKAFTGSVTGYRLFEKESGGSGG
SWSHPQFEK*

Figure S13 | Amino acid sequence of the LUCOSR-base protein. His-tag (pink), SB2 (dark yellow), LB (cyan), protein G domain (red), amber stop codon (yellow), the restriction sites NheI and SpeI for NT-proBNP peptide incorporation (teal and dark red, respectively), mNeonGreen (green), SB1 (dark blue) and Strep-tag (gray). The cysteine used for maleimide coupling is shown in bold and underlined. For the development of the higher affinity SB LUCOSR variant, SB1 and SB2 were mutated to VTGYRLFEKIS and VTGYRLFEKES, respectively.

TSETSGLQEQRNHLQGKTG

Figure S14 | Amino acid sequence of the NT-proBNP immunogenic peptide that was incorporated in LUCOSI. The SpeI (teal) and AgeI (dark red) restriction sites were used to incorporate the peptide into the LUCOSI-base protein plasmid (Figure S9). Leucine 162 that was mutated to an alanine is shown in yellow. The peptide between the two restriction sites was used as target in the NT-proBNP LUCOS titrations.

ASETSGLQEQRNHLQGKTS

Figure S15 | Amino acid sequence of the NT-proBNP immunogenic peptide that was incorporated in LUCOSR. The NheI (teal) and SpeI (dark red) restriction sites were used to incorporate the peptide into the LUCOSR-base protein plasmid (Figure S10). Leucine 162 that was mutated to an alanine is depicted in yellow. The peptide between the two restriction sites was used as target in the NT-proBNP LUCOS titrations.

Supporting references:

(1) Geertjens, N. H. J.; de Vink, P. J.; Wezeman, T.; Markvoort, A. J.; Brunsveld, L. Straightforward Model Construction and Analysis of Multicomponent Biomolecular Systems in Equilibrium. *RSC Chem. Biol.* **2023**, *4*, 252-260.

(2) Krishnamurthy, V. M.; Semetey, V.; Bracher, P. J.; Shen, N.; Whitesides, G. M. Dependence of Effective Molarity on Linker Length for an Intramolecular Protein-Ligand System. *J. Am. Chem. Soc.* **2007**, *129* (5), 1312–1320.

(3) Kjaergaard, M.; Glavina, J.; Chemes, L. B. Predicting the Effect of Disordered Linkers on Effective Concentrations and Avidity with the "C Calculator" App. In *Methods in Enzymology*; Academic Press, **2021**, *647*,145–171.

(4) Marqusee, S.; Baldwin, R. L. Helix Stabilization by Glu-...Lys+ Salt Bridges in Short Peptides of de Novo Design. *Proc. Natl. Acad. Sci. U. S. A.* **1987**, *84* (24), 8898–8902.
